# Phylogenetic and coalescent analysis of three loci suggest that the Water Rail is divisible into two species, *Rallus aquaticus *and *R. indicus*

**DOI:** 10.1186/1471-2148-10-226

**Published:** 2010-07-23

**Authors:** Erika S Tavares, Gerard HJ de Kroon, Allan J Baker

**Affiliations:** 1 Department of Natural History, Royal Ontario Museum, 100 Queen's Park, Toronto, Canada; 2 Havendijk 56,4201 XB 56, The Netherlands; 3 Department of Ecology and Evolutionary Biology, University of Toronto, Toronto, Canada

## Abstract

**Background:**

Water Rails (*Rallus aquaticus*) inhabit fragmented freshwater wetlands across their Palearctic distribution. Disjunct populations are now thought to be morphologically similar over their vast geographic range, though four subspecies had been recognized previously. The fossil record suggests that Water Rails (*R. aquaticus*) were already spread across the Palearctic by the Pleistocene ~2 million years ago, and the oldest fossil remains thought to be closely related to the common ancestor of water rails date from the Pliocene.

**Results:**

To investigate population structure in Water Rails at the genetic level we sequenced three independent loci: 686 base pairs (bp) of the mitochondrial DNA *COI *barcode; 618 bp of the intron *ADH5*; and 746 bp of the exon *PTPN12*. Phylogeographic analysis revealed that Water Rails breeding in eastern Asia (*R. a. indicus*, also known as the Brown-cheeked Rail) are strongly differentiated from the Water Rails in Western and Middle Asia and Europe (*R. a. aquaticus *and *R. a. korejewi*). The Kimura 3-parameter plus Gamma *COI *genetic distance between these two geographic groups was > 3%, and they differed by 18 diagnostic substitutions commensurate with differences between recently diverged sister species of birds. In spite of the low number of variable sites, the two nuclear loci supported this split. We estimated the split of the Brown-cheeked Rail and the Water Rail to have occurred ~534,000 years ago (95% CI 275,000-990,000 years ago). Fragmentation of the widespread ancestral population and eventual speciation of water rails is likely attributable to vicariance by a barrier formed by glacial cycles, continuous uplift of the Tibetan Plateau and increased sedimentation in deserts in southern Asia that originated in the Miocene.

**Conclusions:**

Water Rails from East Asia were genetically differentiated from the ones breeding in Europe and Western to Middle Asia. Most of the genetic signal was from mitochondrial *COI*, and was corroborated by polymorphic sites in the two nuclear loci we employed. The split between these two lineages was estimated to occur in the Middle Pleistocene, when populations were isolated in disjunct wetlands with little or no gene flow. Independent evidence from differences in morphology and vocalizations in concert with genetic differentiation and a long history of isolation support recognition of the Brown-cheeked Rail breeding in East Asia as a separate species, *R. indicus*. The use of several independent loci is invaluable in inferring species trees from gene trees and in recognizing species limits.

## Background

The use of genetic data as a complement to morphological and behavioral data is now common in addressing the problem of species delimitation. Although single mitochondrial genes such as COI used in DNA barcoding have proved highly effective in species delimitation in animals and plants [1,2], it is important test hypotheses of putative species with independent nuclear loci. For recently diverged species, lineage sorting can be incomplete and widespread in genomes, but new coalescent methods exist that can help to detect signals of species even before lineages have become reciprocally monophyletic [3]. Although gene trees can be discordant and complicate the recovery of species trees, they can also be concordant and provide strong support for species delimitation.

**Figure 1:**
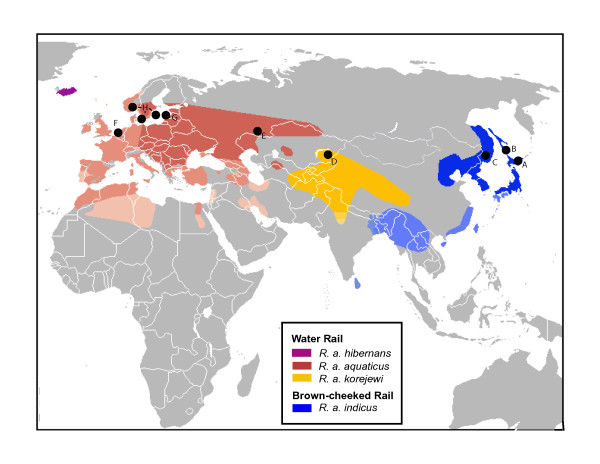
**Distribution map of Brown-cheeked and Water Rails**. Map showing the geographic range of breeding populations of Brown-cheeked and Water Rails [58] and sample localities. Lighter colors correspond to wintering grounds; medium tones to all seasonal grounds; darker colors correspond to breeding grounds. Locality codes are as indicated in Table 1.

One example of taxonomic uncertainty in recently diverged lineages is provided by Water Rails (*R. aquaticus*), which are distributed widely in the Palearctic wetlands from Iceland to Japan, and occupy a range of approximately 10 million km^2^. The global population is thought to be large, with an estimated 290,000-730,000 individuals in their European distribution [4]. Water Rails are confined to wetlands, which have contracted in recent history, and thus their populations are now strikingly fragmented (Figure 1). Further distributional complexity arises because southern and western European populations are mainly sedentary, whereas northernmost European and East Asian populations migrate between northern breeding grounds and southern wintering areas [5].

**Table 1:** Sample sizes

Locality (Abbreviation)		Locality code	Subspecies	*COI*	*ADH5*	*PTPN12*	Combined dataset
**Brown-cheeked Rails**							
East Asia Islands	Hokkaido: Shunkunitai	A	*R. a. indicus*	14	14	14	14
	Russia: Sakhalinskaya Oblast	B	*R. a. indicus*	3	3	3	3
East Asia Continent	Russia: Spasskiy Rayon	C	*R. a. indicus*	3	3	3	3
**Water Rails**							
East Kazakhstan	Lake Alakol	D	*R. a. korejewi*	20	16	16	16
West Siberia	Kargat/Tsjoelim delta	E	*R. a. aquaticus*	17	12	13	12
Europe	Netherlands: Vuren and Zuilichem	F	*R. a. aquaticus*	4	4	3	3
	Latvia: Lakes Engure and Pape	G	*R. a. aquaticus*	8	7	7	7
	Falsterbo, Himlean, Etelhem	H	*R. a. aquaticus*	4	-	-	-
				73	59	59	58

Samples sizes of rails analyzed in this study.

Taxonomic uncertainty exists about the number of species or subspecies that might be recognized in the water rails complex. Some authors suggested that water rails form a superspecies complex with the Kaffir Rail (*R. caerulensis*) and Madagascar Rail (*R. madasgascariensis*) due to similarities in size, coloration, and the startling calls they share [6-8].

Water Rails had previously been placed in four subspecies differentiated by morphological variation and geographic range: *R. a. aquaticus *in Europe including the British Isles, Northern Africa to North-western Asia; *R. a. hibernans *in Iceland; *R. a. korejewi *in Western to Middle Asia, and *R. a. indicus *in Eastern Asia, including Japan [5,7]. However, recent morphological studies supported subspecies status (*R. a. indicus*) for Eastern Asian birds [9,10], but could not detect differences between the other three subspecies. *R. a. korejewi *and *R. a. hibernans *differ by small variations in external plumage and measurements that overlap gradually from west to east. The Icelandic population of *R. a. hibernans *became extinct about 1965 due to widespread draining of wetlands and predation by Mink (*Mustela vison*), and now non-breeding birds probably from Scandinavia migrate to Iceland in autumn and winter [11]. Birds from East Asian populations are distinguished from those in West and Middle Asia and Europe by proportionally larger dimensions, less sexual dimorphism is size, less contrastingly banded flanks, black-and-white-barred under tails coverts (white in the other populations), darker tip of the neck and breast feathers, higher percentage of barred outer coverts in the wing, and a whiter supercilium above a brown eye-stripe [9,12]. A recent classification therefore referred to this lineage with a distinct common name as the Brown-cheeked Rail (*R. a. indicus*), reflecting differences from the other Water Rail subspecies (*R. a. aquaticus *and *R. a. korejewi*) [13], and hereafter we use these vernaculars and refer to them collectively as water rails.

The oldest fossil remains of a rail which is the closest relative to the ancestor of all water rails are phalange bones found in caves in the Carpathian basin dated from the Pliocene (5.3-1.8 Million Years; International Stratigraphic Chart, ISC) [14,15]. More recent fossils from the Upper Pleistocene suggest that water rails were already widely distributed two million years ago; fossils have been found in Ireland, United Kingdom, Belgium, France, Italy, Cyprus, Germany, Poland, Austria, Croatia, Bosnia-Herzegovina, Hungary, Czech Republic, Ukraine, Romania, Greece, Jordan, Israel, China and Japan [16]. Climate and environmental changes associated with the last four ice ages, along with tectonic activities that occurred in Europe and Asia, could potentially have had a great impact on water rail populations. The association of particular historical events with population differentiation and possibly speciation requires inference of the species tree, divergence times and levels of gene flow.

In the present study we investigate the phylogeny and population structure of the water rail species-complex using three independent loci, including the COI DNA barcode region. Specifically, we investigate whether genetic evidence supports the Brown-cheeked Rail and Water Rail as separate species, the number of subspecies that should be recognized, and whether phylogeographic patterns within the water rail complex relate to known historical events that may have structured populations genetically.

## Results

### Genetic variation and base composition

The numbers of base pairs (bp) sequenced for *COI*, *ADH5*, and *PTPN12 *were respectively 686, 618 and 746, yielding a combined data set of 2,050 bp. The number of individuals sequenced for each population varied, but sequences for all three genes were determined for 58 water rails, and two Virginia rails (*Rallus limicola*, Table 1). Translation of the protein-coding genes *COI *and *PTPN12 *did not reveal stop codons or frame-shift mutations, and third codon positions were more variable than first and second codon positions as expected in functional genes. Three indels of one or two bases were observed in the *ADH5 *intron. Polymorphism due to heterozygosity was observed in the nuclear partitions *ADH5 *and *PTPN12*. The average base composition among water rails for each gene partition was 26.1% A, 33.4% C, 16.7% G, 23.8% T for *COI*; 25.8% A, 20.1% C, 22.3% G, 31.8% T for *ADH5*; and 29.1% A, 24.2% C, 23% G, 23.7% T for *PTPN12*. Best-fit models of nucleotide substitution selected with the Akaike information criterion (AIC) were unequal-frequency Kimura 3-parameter plus Gamma (K81uf + G = 0.1704) for *COI*, and Hasegawa-Kishino-Yano (HKY) for *ADH5 *and *PTPN12*.

**Figure 2:**
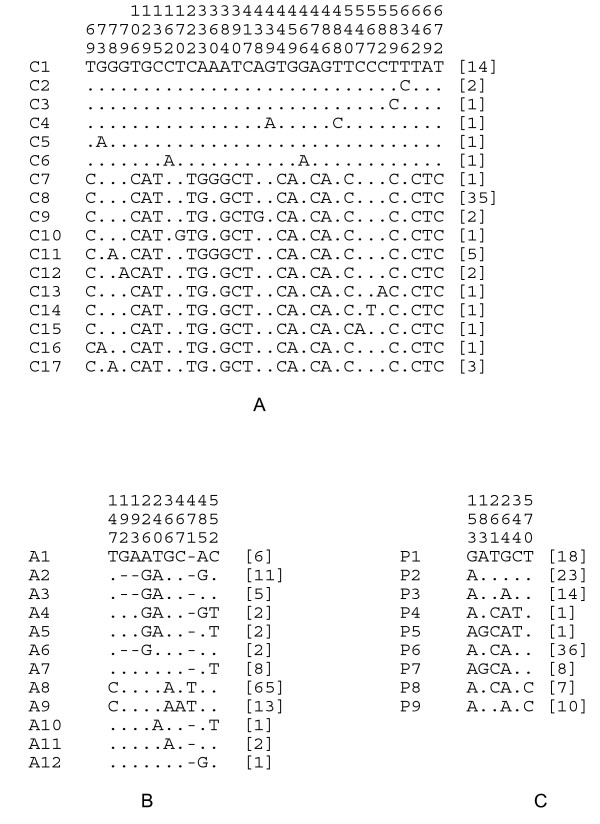
**Haplotypic and allelic variation in Brown-cheeked and Water Rails**. Variable nucleotide positions found in a) 686 bp of the mitochondrial *COI*; b) 618 bp of the intron *ADH5*; and c) 746 bp of the nuclear exon *PTPN12*. Numbers above sequences correspond to nucleotide positions in the sequences. The frequency of haplotypes or alleles is shown in parentheses. Dots indicate nucleotide matches with the first sequence.

**Figure 3:**
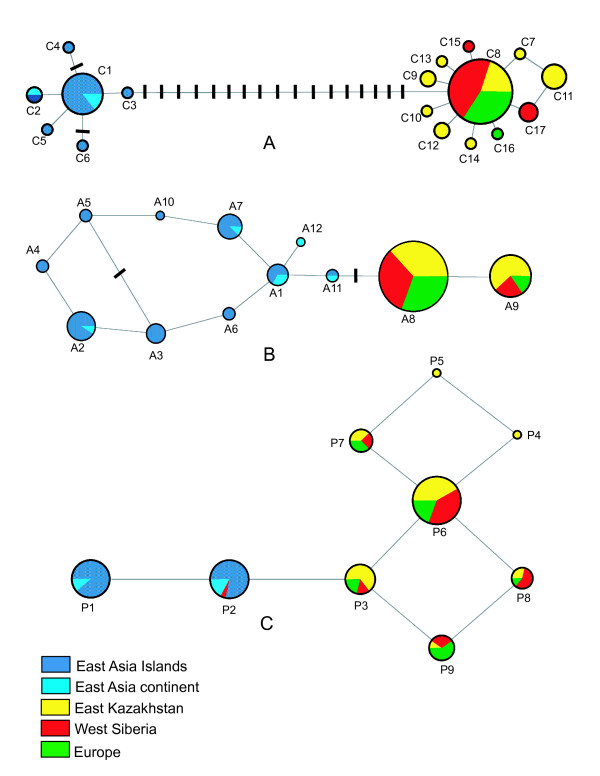
**Median-joining networks**. Median-joining networks connecting haplotypes found in populations of Brown-cheeked and Water Rails for three molecular markers a) *COI*; b) *ADH5*; and c) *PTPN12*. Circles represent individual haplotypes listed in Figure 1. The sizes of the circles are proportional to the number of individuals (in A), or alleles (in B and C) found to possess each haplotype, and the color code corresponds to sample locality. Undashed network branches correspond to single nucleotide substitution between the adjacent haplotypes, and dash marks along branches correspond to additional mutational steps connecting the adjacent haplotypes.

A total of 17 unique haplotypes defined by 32 variable sites was identified in sequences from mitochondrial *COI *of 73 specimens of Brown-cheeked and Water Rails (Figure 2A). Sequences from phased genotypes of *ADH5 *and *PTPN12 *revealed 10 and six variable sites identifying 12 and nine unique haplotypes in each nuclear gene, respectively (Figure 2B, C). The Brown-cheeked Rail and the Water Rail did not share alleles for the three genes studied, except for one allele (P2) in the most slowly evolving gene, *PTPN12*, which could reflect retained ancestral polymorphism (Figure 3). West and Middle Asian and European populations from East Kazakhstan, West Siberia, and Europe were included in the Water Rail clade, they shared haplotypes in the three genes, and showed the highest haplotype diversity in the faster mitochondrial gene *COI*, as expected. Moreover, a single haplotype (C8) was the only one common to all populations in the western part of the Water Rail distribution. Singleton *COI *haplotypes differed from the most common haplotype (C8) by one or two mutational steps (Figure 3). The number of mutational steps separating the Brown-cheeked Rail and Water Rail was 18 and two, for *COI *and *ADH5*, respectively. With the exception of one shared haplotype, one mutational step separated the two groups of water rails at *PTPN12*. Corresponding corrected genetic distances, calculated using the best-fit model for each partition were 3.3%, 0.6%, and 0.3%, for *COI*, *ADH5 *and *PTPN12*, respectively.

### Phylogenetic analysis and relationship among breeding populations

Monophyletic groups of Brown-cheeked Rails and Water Rails were recovered in ML and Bayesian analyses of *COI *(N = 73) and *ADH5 *(N = 59), with a posterior probability of 1 for clusters in *COI *(Additional file 1). Analyses of *PTPN12 *(N = 59) revealed a monophyletic group composed of all sampled individuals of the Brown-cheeked Rail, but node support was lower (0.89, Additional file 1). Reciprocal monophyly of Brown-cheeked Rails and Water Rails was strongly supported in a Bayesian analysis of the three genes combined (Additional file 2). The chance occurrence of reciprocal monophyly between the Brown-cheeked Rail and Water Rail was rejected (p = 1.9 × 10^-17^, group a = 20 individuals, group b = 38 individuals).

**Figure 4:**
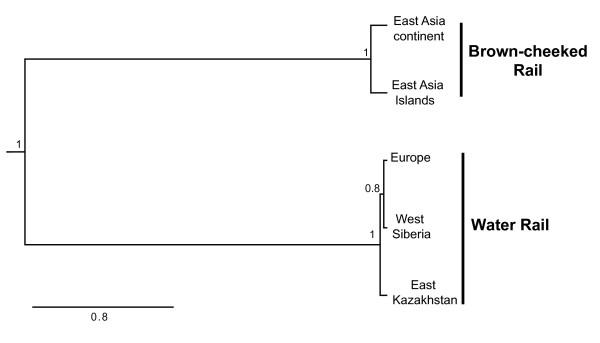
**Species tree of Brown-cheeked and Water Rails**. Species tree estimated in *BEAST based on 686 bp of *COI *sequences, 618 bp of the intron *ADH5*, and 746 bp of the exon *PTPN12 *with phased genotypes for the nuclear partitions. The topology is shown as a chronogram, and numbers at nodes correspond to their posterior probabilities.

The species tree estimated with *BEAST recovered the Brown-cheeked Rail and Water Rail as reciprocally monophyletic groups with posterior probabilities of 1 (Figure 4). Within these clades the relationships among the sampled populations were not resolved. This reflects the high number of shared alleles among the populations of both Water Rails and Brown-cheeked rails, but not between them.

### Population structure and coalescent analyses

Sequence data sets for all populations had nonsignificant values (p > 0.10) of Tajima's D values and Fu and Li's D* and F* statistics, suggesting all three genes are selectively neutral. Therefore, it is appropriate to use them to estimate gene flow and other parameters with neutral coalescent methods.

Analyses of the genotypes at the two nuclear loci using Structure supported two major clusters corresponding to the population samples of the Water Rail and the Brown-cheeked Rail. The posterior probability of this run was 0.57, versus 0.43 for the model assuming 3 populations (-ln = 276.4 versus -ln = 276.7). However, the model with two populations performed better than the one with three, with estimated mean population assignment probabilities of individuals in populations 1 and 2 of 0.991 (0.954,1.0) and 0.990 (0.948, 1.0), respectively. The model with three populations performed poorly in assigning samples of the Water Rail to two clusters, the highest population assignment probability for membership of an individual to clusters 2 and 3 being only 0.571 (0, 1.0).

Both nuclear loci showed recombination by the four-gamete criterion, *ADH5 *between sites 195 and 469, and *PTPN12 *between sites 238 and 344. However, the program IM used to estimate gene flow assumes no within-locus recombination, so we split each locus into two blocks of sequence that did not show evidence of recombination [17]. Blocks were from characters 1 to 195 and 196 to 618 for *ADH5*, and 1 to 343 and 344 to 746 for *PTPN12*. Each of these blocks was analyzed in IM as different loci.

**Figure 5:**
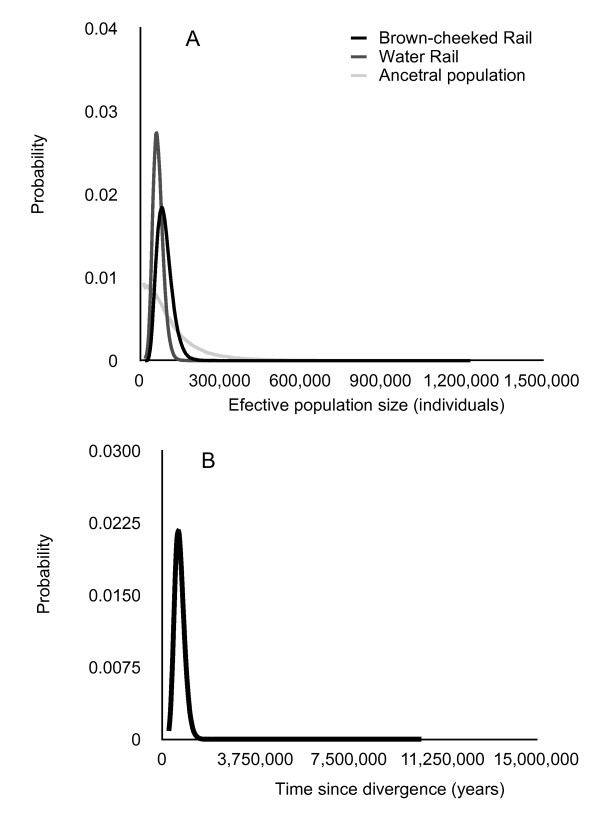
**Demographic estimates for the Brown-cheeked and Water Rail assuming a model of isolation without gene flow**. a) Effective population size for the Brown-cheeked Rail, Water Rail, and their ancestral population; b) Divergence time between the Brown-cheeked Rail and Water Rail.

The coalescent analysis assuming the less parameterized isolation-without-migration model had marginally significantly lower log likelihood than the isolation-with-migration model (-2[lnL0-lnL1] = 7, df = 2, p = 0.03). The latter suggested no gene flow between populations of the Brown-cheeked and Water Rail after their divergence; values of m_1 _= 0.015 (95% CI 0.005-1.04) and m_2 _= 0.405 (95% CI 0.045-1.385) were very low and the lower 95% credibility intervals approached zero. Therefore we interpreted the coalescent parameters of divergence time and effective population size from the analysis using the model of isolation-without-migration. This less parameterized model also allowed divergence time to be estimated more precisely (Figure 5), suggesting the split between Brown-cheeked and Water Rails occurred ~534,000 years ago (95% CI 275,000-990,000 years). The effective population size estimated for the Brown-cheeked Rail was 77,000 (95% CI 38,000-147,000), which was similar to the values estimated for Water Rails (56,000; 95% CI 30,000-99,000; Figure 5). Effective size of the ancestral population could not be estimated properly with three loci, and sorting of alleles into reciprocally monophyletic groups (Figure 5).

## Discussion

### Species delimitation is supported by genetics, morphology and vocalizations

Analysis of DNA sequences indicated unambiguously that the birds from insular and continental locations in East Asia (referred to as *R. a. indicus *or Brown-cheeked Rails) are clearly differentiated from the groups sampled further west from breeding populations in Europe, West Siberia, and Kazakhstan (originally recognized as *R. a. aquaticus *and *R. a. korejewi*). The overall Kimura 3-Parameter genetic distance between Water Rail and Brown-cheeked Rail clades for *COI *was > 3% including 18 fixed nucleotide substitutions. This number of substitutions falls well within the range expected between closely related species. Of 59 sister species-pairs of birds surveyed with the same COI barcode sequence 22 were separated by fewer than 18 diagnostic differences [18].

Sequences from the *COI *DNA barcode region indicated a clear subdivision that was corroborated with two nuclear genes, with haplotypes mostly confined to each clade, except for one allele in the more slowly evolving nuclear gene *PTPN12*. The most likely explanation in this case is retention of ancestral polymorphism but not recent hybridization, as no other allele was shared in the faster evolving *ADH *intron or *COI*. The use of multiple genes not only increased the support for species delimitation, but it also provided more information to improve the precision of the coalescent estimates of divergence time and assessment of the degree of genetic isolation. Coalescent analysis suggested that divergence with little or no gene flow occurred between the two groups in the Middle Pleistocene. These observations are in accordance with morphological differences between Brown-cheeked Rails and Water Rails observed in comprehensive sampling of museum skins. Eastern birds are sexually monomorphic in size, have proportionally larger dimensions, pinkish cinnamon and pale buff chin and throat-feathers, longer neck and breast feathers, and black-and-white-barred under-tail coverts [9]. Additionally, the two forms have different call types: the complex call of the Brown-cheeked Rail has a lower pitched growl and a distinctly shorter duration than in the Water Rail, which seems to have biological significance due the lack of response to each other's calls in playback experiments [19] (Table 2).

**Table 2:** Divergent traits between the Water Rail and Brown-cheeked Rail (adults)

Traits	Water Rail (*Rallus aquaticus*)	Brown-cheeked Rail (*R. indicus*)
Sexual dimorphism in body size	Present	Absent
Body size	Smaller	Larger
Primary measurements	Smaller	Larger
Primary-index	Smaller	Larger
Bill length	Longer and finer	Shorter
Supercilium	Darker	Lighter (looks to conspicuous/marked)
Chin and throat feathers	White or light gray	Pinkish cinnamon
Vane tips of neck-and-breast feathers	Pale/gray	Brownish
Under tail coverts	White	Deep black-and-white barred
Outer coverts of the wing	Less frequently barred	More frequently barred
Pitched growl in call	Low	Very low
Call duration	Longer	Shorter
Length of eggs	Smaller	Larger

Trait variation observed between adults of the Water Rail and Brown-cheeked Rail [7,10,15].

A unified species concept suggests treating independently evolving metapopulation lineages as different species, if independent lines of evidence are available to support their reciprocal isolation [20]. Hence, we support raising the Brown-cheeked Rail to full species status, *Rallus indicus*, since genetic evidence and differences in morphology and vocalizations indicate this lineage is evolving independently from populations of the Water Rail (*R. aquaticus*).

### Vicariance and biogeography

According to our analysis the Brown-cheeked Rails and Water Rails diverged with little or no gene flow about 530,000 years ago, and thus fit a model of allopatric speciation where a geographic barrier prevented contact between the two groups. Prior to this the fossil record indicates that water rails were widespread in Eurasia including Japan from at least the beginning of the Pleistocene (~ 1.8 Million years, ISC). By the end of the Pliocene, the climate was considerably different from that of today, coniferous forests covered much of the modern tundra zone, and grasslands extended into mid- and low-latitudes [21]. The distribution of wetlands in this moister climate was likely more continuous, and the dispersal of water rails in Eurasia could have occurred via multiple routes.

The Pleistocene was marked by repeated glacial cycles [22,23] that at their maxima would have prevented passage of several populations through Siberia, and around high mountain chains due to the higher extension of permafrost [24]. The glaciers also would have tied up a considerable volume of water, reducing precipitation and contributing to the extension of Asian deserts such as the Taklamakan and Gobi, consistent with records in loess deposits in China and Tajikistan [25]. The continuous uplift of the Himalayan mountain chain, which doubled its height during the period, was not only a barrier on its own, but is also believed to have contributed to the extension of the arid areas by creating a barrier for the monsoon winds [25,26]. These events limited the availability of wetlands, and likely forced water rails south to less continuous wetland environments east and west of the arid spots. Fragmentation of the widespread ancestral population and eventual speciation of water rails is thus attributable to this vicariance event. Currently, Brown-cheeked Rail and Water Rail populations are isolated on opposite sides of the Yablonovy, Stanovoy and Himalaya mountain chains, the Mongolian steppes, and the Gobi desert [19].

### Lack of population structure within species

Despite the sparse sampling across their range in Eurasia, we did not detect obvious structuring of breeding populations of the Water Rail from Europe, West Siberia and Kazakhstan. This supports lumping of *R. a. aquaticus *from Europe and West and Middle Siberia with the disjunct population of *R. a. korejewi *from Kazakhstan in one subspecies, consistent with their lack of morphological differentiation [5]. Water rails are known to have an erratic migration pattern, and good flight capabilities, which could allow them to fly long distances to find alternative shallow water habitats in times of instability [9]. Long distance gene flow could explain lack of population structure in birds from the west. However, low frequency haplotypes in COI are not shared between different breeding populations, indicating that interpopulation gene flow is not extensive enough to spread these more recently evolved mutant alleles. A star-burst distribution of haplotypes in the water rail lineages (Figure 3A) suggests a population contraction occurred, which would tend to erase less frequent haplotypes, and then the population expanded in size [27]. Using 534,000 years ago time of divergence of the two species as a calibration in the species tree estimated with *BEAST, we dated the expansion of the populations in both species at approximately 10,000 years ago. This recent expansion was likely from a refugial population that survived the Last Glacial Maximum. These hypotheses need to be addressed in the future with more extensive sampling of individuals and more rapidly evolving nuclear loci.

## Conclusions

Our analysis with three genetic markers is consistent with morphological and call differences that collectively support suggestions that the Water Rail is divisible into two species, the Brown-cheeked Rail (*R. indicus*), including breeding populations of East Asia, and the Water Rail (*R. aquaticus*), including populations from West Asia and Europe. These lineages were estimated to have split in the Middle Pleistocene (~534,000 years ago) when wetlands became more disjunct, restricting gene flow and eventually isolating them in allopatric populations.

## Methods

### Taxon sampling

Blood samples from apparently non-related individuals of breeding populations of Brown-cheeked Rails and Water Rails (Figure 1) were collected in different localities within their geographic range in Europe and Asia, and were grouped according to their breeding ground proximity and wintering migration area (Table 1, see locality information per sample in Additional file 3). COI sequences of birds from four additional samples in three different localities in Europe (Falsterbo, Himlean, Etelhem) were obtained from the DNA barcoding database [28,29] (Table S1). A drop of blood was taken from each bird by puncture of the brachial wing vein and mixed in 0.1 M EDTA and 1 mL 80% ethanol. Sample sizes varied for the different loci (see Table 1), with the biggest sample obtained for the fastest evolving gene studied, *COI *(73 individuals), which allowed better interpretation of historical population demography. The combined data set for the three genes included 58 individuals. Two individuals of Virginia rail (*R. limicola*) were used as outgroups. The *COI *sequences generated and used in this work are deposited in the project "Royal Ontario Museum-Rails" in the Completed Projects selection of the Barcode of Life Data System (BOLD) [30], and GenBank (Accession numbers GU097202 - GU097266 and HM474036-HM474041, Additional file 3). Nuclear gene sequences and haplotypes are deposited in GenBank (GU097202-GU097249) and correspond to individuals and sample sites in Additional file 3.

### DNA extraction and sequencing

DNA was extracted by a membrane purification procedure in glass fiber-filtration plates (Acroprep 96 Filter Plate-1.0 μm Glass, PALL Corporation) [31]. Sequences were obtained from three independent loci (Table 1): 1) the 5'end of the mitochondrial gene cytochrome oxidase I (*COI*) used in DNA barcoding [1]; 2) intron 5 of the nuclear gene alcohol dehydrogenase-I (*ADH5*) [32]; and c) a segment of the nuclear gene Tyrosine-protein phosphatase non-receptor type 12 (*PTPN12*) [33]. Polymerase Chain Reaction (PCR) amplifications were performed in 12.5 μL reactions in a buffer solution containing 10 mM Tris-HCl (pH8.3), 50 mM KCl, 2.5 mM MgCl_2_, 0.01% gelatin, 160 μg/ml bovine serum albumin (BSA) [34], 0.4 mM dNTPs, 0.2 μM of each primer, 1 U *Taq *Polymerase (*Invitrogen*) and 20-25 ng of DNA. Cycle conditions for *COI *were an initial denaturation at 94°C for 5 min, 36 cycles of 94°C for 40 sec, 50°C for 40 sec and 72°C for 1 min, and a final extension at 72°C for 7 min. Touchdown cycle conditions were used for nuclear markers, as follows: an initial denaturation at 94°C for 3 min, 15 cycles of 94°C for 1 min, 65-55°C (decreasing 1°C per cycle) for 35 sec, and 72°C for 1 min, followed by 26 cycles of 94°C for 1 min, 50°C for 35 sec, 72°C for 1 min, and a final extension at 72°C for 2 min. Primers used to amplify and sequence *COI *were LTyr (forward - TGTAAAAAGGWCTACAGCCTAACGC, Oliver Haddrath, pers. comm.) and COI748HT (reverse - TGGGARATAATTCCRAAGCCTG) [18]; for *ADH5 *we used ADH5F (forward-TCTGTTGTCATGGGCTGCAAG) [32] and ADH6R (reverse-TCCAAAGACGGACCCTTTCCAG, 31) [32]; and for *PTPN12 *we used PTPN12f1 (forward-AGTTGCCTTGTWGAAGCCCGCATACA) [33] and PTPN12_r6 (reverse-CTRGCAATKGACATYGGYAATAC) [33]. PCR products were purified by excising bands from agarose gels and centrifuging each through a filter tip [35]. Sequences were obtained on an ABI3100 (*Applied Biosystems*).

### Sequence statistics, genetic diversity, and phylogenetic analysis

Sequences were checked for ambiguities, and alignments were assembled in Sequencher 4.1.2 (GeneCodes Corp., Ann Arbor, Michigan) and MacClade 4 [36]. Single nucleotide polymorphisms (SNPs) derived from gene sequences of nuclear loci (*ADH5 *and *PTPN12*) were assigned to a single chromatid statistically in PHASE 2.1 [37], and the associated SNPs were referred to as haplotypes [37]. DNAsp 4.90.1 [38] was used to estimate haplotype diversity for each independent locus and to generate haplotype matrices. Median-joining networks to show relationships among nuclear alleles and mtDNA haplotypes were built in Network 4.1 [39].

Base composition and genetic distances among individuals were calculated with PAUP* 4b10 [40]. The best-fit models of nucleotide evolution for each gene partition and for the combined data set were selected with the Akaike information Criterion (AIC) in Modeltest 3.7 [41]. To verify if independent partitions were supporting congruent phylogenetic signals, Maximum Likelihood (with one allele per bird), and Bayesian analysis (of all alleles) of each gene tree and of the concatenated data set (including only individuals sampled for all three genes) were compared. Maximum likelihood analyses were performed in PHYML [42] with 100 bootstrap replications for node support, using the best-fit models estimated with AIC in Modeltest. Bayesian analyses were performed by Markov Chain Monte Carlo (MCMC) in MrBayes 3.2 [43] in two simultaneous independent runs of 5 million generations each, with one cold and three heated chains and sampling once every 1000 trees. The best-fit models were used for each partition, but the values of the model parameters were jointly inferred in the run. Posterior probabilities of the nodes were computed across the sampled trees after burn-in, which was determined by convergence of the likelihood scores.

Compound diagnostic characters are a valuable source of information to diagnose species [18,44], so we filtered all the variable characters for each gene partition in PAUP*, and the selected the fixed substitutional differences between each highly supported clade within water rails. The test of chance occurrence of reciprocal monophyly was applied to the highly supported monophyletic clades (Bayesian posterior probabilities > 0.95) recovered with the concatenated Bayesian analyses to try to distinguish if they likely resulted from random branching in a single population or if they might represent distinct taxonomic entities [45].

To estimate the species and population tree we used the program *BEAST v1.5.4 (Bayesian Evolutionary Analysis Sampling Trees) [46]. This method employs substitution models used in traditional phylogenetics, but also uses coalescent theory to provide joint inferences of a species tree topology, divergence times, population sizes, and gene trees from multiple genes sampled from multiple individuals across a set of closely related species [46]. As we are testing the phylogenetic relationships among populations of Brown-Cheeked and Water Rails, the haplotypes of the three genes (Table 1) were grouped in five different trait sets, defined by the localities of the breeding populations. The best-fit model estimated with AIC in Modeltest were set for each partition a priori, and the model parameters were estimated with the tree topologies in the analysis. In addition to the substitution model, the clock model and tree topologies were estimated independently for each gene. We set the clock model as a strict clock because the two species are very closely related. A prior for the rate of substitution of *COI *was set at 0.772%/lineage/Myr, based on the mean for the NeoAves clade derived in a mitogenomic timescale for birds [47]. Setting this rate for the mitochondrial partition allowed us to estimate the rate of substitution of the two nuclear partitions. Two independent simultaneous runs of 100,000,000 generations were performed, sampling once every 1,000 trees. Posterior probabilities of the nodes were computed for all Bayesian analyses across the sampled trees after burn-in. The number of generations required to reach stationarity of the posterior distribution was determined by examining marginal probabilities plotted as a time series in TRACER v1.5 [48]. The burn-in period was set as 30,000 trees (30,000,000 generations). The effective sample sizes (ESS) of parameters of interest (gene trees, species tree, root age) were all above 200.

### Among-population gene flow and coalescent time

Basic coalescent models in population genetics assume independence between loci, and selective neutrality. We therefore used only one mitochondrial gene, in spite of higher genetic variability displayed by this genome, which would provide more variable characters for demographic estimates. *COI *was selected as the mitochondrial gene because in an initial survey for a DNA barcoding project, we observed structured variability among Brown-Cheeked and Water Rails at this locus. Two nuclear loci were also selected because they potentially can provide evidence for subdivision in genes with independent histories. The nuclear genes *PTPN12 *and *ADH5 *are located in chromosomes 1 and 4, respectively in *Gallus gallus *[49]; they also likely segregate independently in rails because bird macrochromosomes generally have high synteny [50,51]. To verify if the sequences conform with neutral expectations of nucleotide substitution, we computed Tajima's D-value and Fu and Li D* and F* values [52] for each gene using DNAsp 4.90.1 [38].

Population structure using phased genotypes of the two nuclear alleles were estimated in Structure 2.3.1 [53,54]. For this purpose, we used the output of PHASE, which codes sequence information in numbers. The method assumes a model in which K populations are characterized by a set of allele frequencies at each locus. Individuals are assigned probabilistically to populations in a way that approximates the allele frequencies at each locus to Hardy-Weinberg equilibrium. Multiple runs were performed using the admixture model, with values of K varying from 1-5 (due the putative structure of the different breeding populations) for 2,000,000 generations and a burn-in period set to 400,000 generations. The run for each model with the highest likelihood value was selected as the best approximation of population structure in water rails. The posterior probability of each value of K was calculated with Bayes rule.

Divergence time and other demographic quantities of the two well supported clades within water rails (Table 1) were estimated with IM [17]. This program implements a model of isolation with migration, accounting for changes in population sizes and the size of the ancestral population. The program uses a Markov Chain Monte Carlo to estimate jointly the posterior distribution of the model parameters and the demographic quantities N_1_, N_2_, and N_A _(effective sizes of population 1, population 2, and the ancestral population, respectively), t (time since population splitting), m_1_, and m_2 _(migration rate per generation of genes from population 2 to population 1, and from population 1 to population 2, respectively). The fitting of the IM model assumes that the genealogical history of a locus is bifurcating and does not include recombination [17]. To test for recombination in the nuclear partitions, we used the four-gamete test [55] in DNAsp [38]. Two analyses were performed in IM: the first allowing migration between the two populations; and the second assuming the isolation model where migration rates (*m*_1 _and *m*_2_; migration rate scaled by mutation rate) where set to 0. A likelihood ratio test (2 degrees of freedom) was used to verify if the model of isolation-without-migration was significantly different from the more parameter-rich model of isolation with migration. The available option of the HKY model of sequence evolution was adopted for each gene to correct for multiple substitutions at sites. Prior values of mutation rates (μ) for each locus, and generation time, were specified to convert coalescent times to years before present. For mitochondrial *COI*, we used the rate of 0.772%/lineage/Myr estimated for the clade of Neoaves sampled in a mitogenomic timescale for birds [47], which was converted to 5.3 × 10^-6 ^substitutions/locus/year (s/l/y) by multiplying by the number of base pairs of the locus (686), and transforming from million years to years. For the nuclear partitions we used the mean value of the posterior distribution of the rates obtained in *BEAST analysis. The rate estimated for ADH5 was 3.27 × 10^-9 ^substitutions/site/year (s/s/y), and for PTPN12 was 3.13 × 10^-9 ^s/s/y. They were converted to the required per locus rate of the non-recombining blocks used in IM, by multiplying the per site rates by the corresponding number of bases used in each block. Generation time (g) of Brown-Cheeked and Water Rails was set as 2 years [56]. As the method allows input of different sample sizes per locus, the maximum number of individuals that were sequenced for each locus was used. Several preliminary runs were performed in IM with different priors and heating schemes to find the conditions that allowed proper mixing among chains to avoid local optimum parameter values. We also monitored the mixing of the chains by observing the effective sample size (ESS) for each parameter that was estimated. When good conditions were achieved, three runs were performed for each analysis to verify if the estimated parameters were converging to similar results. Final IM analyses were run for 4,600,000 generations after a burn-in of 100,000 steps using geometric heating, with high heating parameters (g_1 _= 0.8 and g_2 _= 0.9), and 25 chains. Priors were set to maximum values for the parameters: *t *(time of split x μ) set as 15; for the run allowing migration, maximum *m*_1 _and *m*_2 _value were set to 10; for the run assuming no gene flow among the populations *m*_1 _and *m*_2 _were set to 0. The minimum ESS values for the parameters estimated in the analysis with and without migration were 352 and 743, respectively. In both cases ESS values for the parameters of interest were well above 50, the minimum value recommended [17].

## Authors' contributions

GdK collected the blood samples in field and drafted the background information; AJB devised the design of the study, EST generated the molecular data and performed the analyses, and the three authors drafted the manuscript. All authors read and approved the final manuscript.

## Supplementary Material

Additional file 1 **Maximum likelihood gene trees for individual gene partitions**. Maximum likelihood tree topology of Brown-cheeked and Water Rails based on a) 686 bp of *COI *sequences, 618 bp of the intron *ADH5*, and 746 bp of the exon *PTPN12*. Scale bars correspond to the expected number of substitutions per site. Numbers at the nodes correspond to Bayesian posterior probabilities > 0.95 (above) and bootstrap proportions above 50% (below). Dots on the branches correspond to the number of fixed substitutions supporting the clade. Individuals sampled are shape-and-shade-coded by sample locality. Click here for file

Additional file 2 **Bayesian Analyses**. Bayesian analysis of Brown-cheeked and Water Rails based on 686 bp of *COI *sequences, 618 bp of the intron *ADH5*, and 746 bp of the exon *PTPN12*. Scale bars correspond to the expected number of substitutions per site. Numbers at the nodes correspond to Bayesian posterior probabilities. Sampled individuals are color-coded by collection locality. Click here for file

Additional file 3 **Specimens Details**. List of specimens used in the study, with detailed identification information, sample locality coordinates, and corresponding nuclear alleles. Click here for file
