# Supplementary material for: Phylogenetic and coalescent analysis of three loci suggest that the Water Rail is divisible into two species, Rallus aquaticus and R. indicus
Source: BMC Evol Biol. 2010 Jul 23;10:226. doi: 10.1186/1471-2148-10-226 (PMC2927924; doi:10.1186/1471-2148-10-226)
Supplement: Additional file 1 — Maximum likelihood gene trees for individual gene partitions. Maximum likelihood tree topology of Brown-cheeked and Water Rails based on a) 686 bp of COI sequences, 618 bp of the intron ADH5, and 746 bp of the exon PTPN12. Scale bars correspond to the expected number of substitutions per site. Numbers at the nodes correspond to Bayesian posterior probabilities > 0.95 (above) and bootstrap proportions above 50% (below). Dots on the branches correspond to the number of fixed substitutions supporting the clade. Individuals sampled are shape-and-shade-coded by sample locality. [file 1471-2148-10-226-S1.PDF]

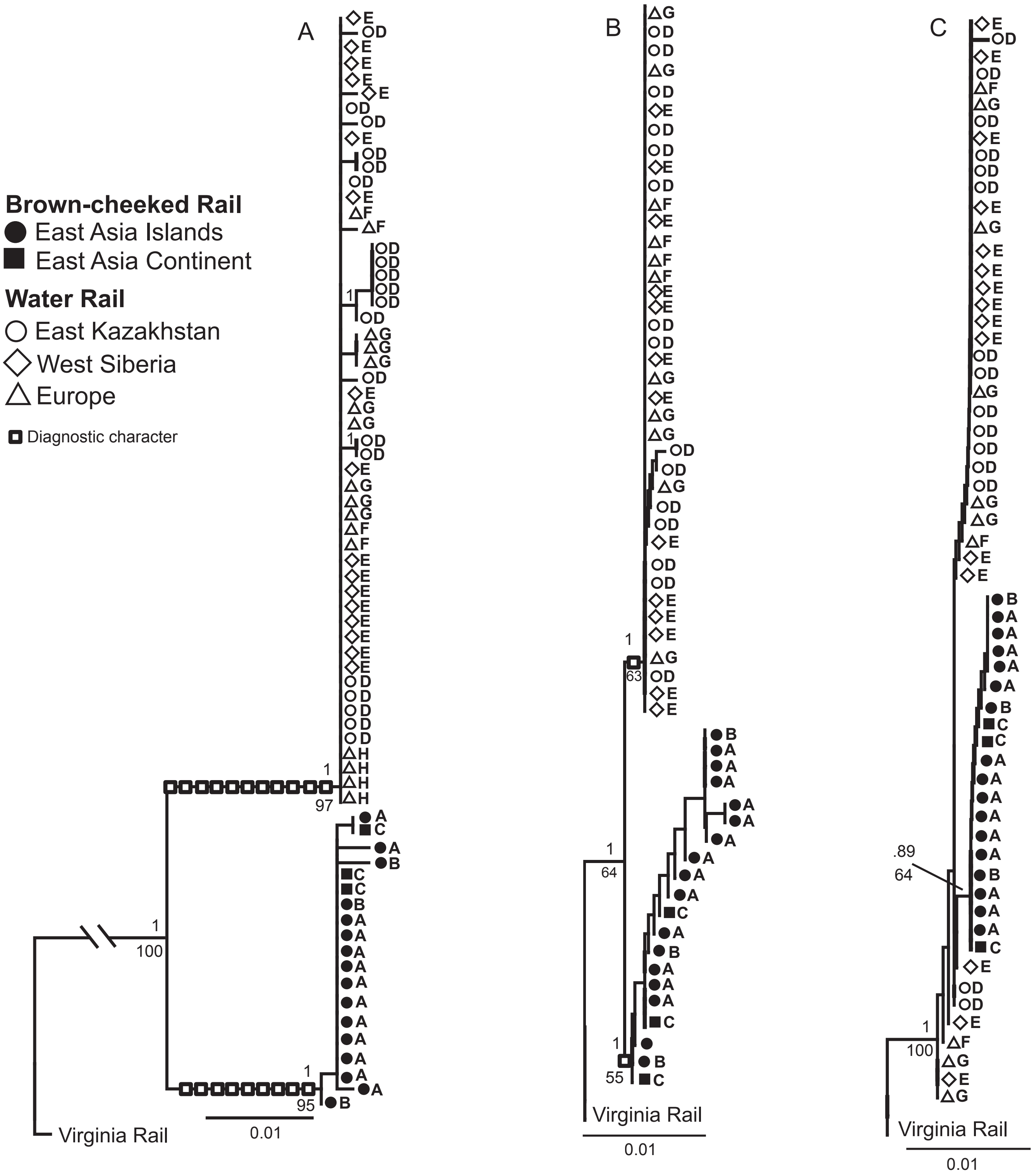
A
B
C
Brown-cheeked Rail
East Asia Islands
East Asia Continent
Water Rail
East Kazakhstan
West Siberia
Europe
Diagnostic character
Virginia Rail
0.01
Virginia Rail
0.01
Virginia Rail
0.01
