# Supplementary material for: Phylogenetic and coalescent analysis of three loci suggest that the Water Rail is divisible into two species, Rallus aquaticus and R. indicus
Source: BMC Evol Biol. 2010 Jul 23;10:226. doi: 10.1186/1471-2148-10-226 (PMC2927924; doi:10.1186/1471-2148-10-226)
Supplement: Additional file 2 — Bayesian Analyses. Bayesian analysis of Brown-cheeked and Water Rails based on 686 bp of COI sequences, 618 bp of the intron ADH5, and 746 bp of the exon PTPN12. Scale bars correspond to the expected number of substitutions per site. Numbers at the nodes correspond to Bayesian posterior probabilities. Sampled individuals are color-coded by collection locality. [file 1471-2148-10-226-S2.PDF]

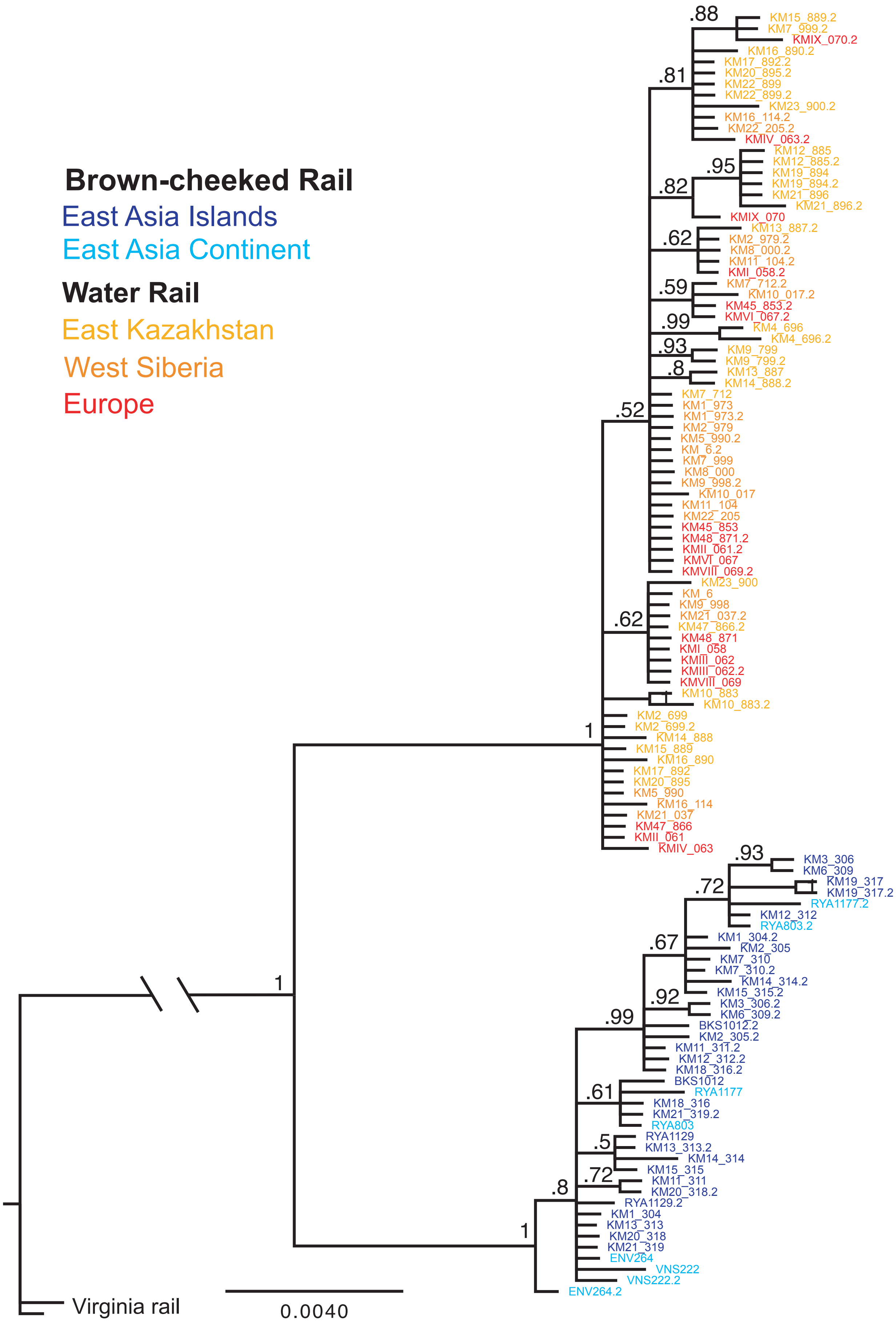
Brown-cheeked Rail
East Asia Islands
East Asia Continent
Water Rail
East Kazakhstan
West Siberia
Europe
.88
KM15_889.2
KM7_999.2
KMIX_070.2
KM16_890.2
.81
KM17_892.2
KM20_895.2
KM22_899
KM22_899.2
KM23_900.2
KM16_114.2
KM22_205.2
KMIV_063.2
.95
KM12_885
KM12_885.2
KM19_894
KM19_894.2
KM21_896
.82
KM21_896.2
KMIX_070
.62
KM13_887.2
KM2_979.2
KM8_000.2
KM11_104.2
KMI_058.2
.59
KM7_712.2
KM10_017.2
KM45_853.2
KMVI_067.2
.99
KM4_696
KM4_696.2
.93
KM9_799
KM9_799.2
.8
KM13_887
KM14_888.2
KM7_712
KM1_973
KM1_973.2
KM2_979
KM5_990.2
KM_6.2
KM7_999
KM8_000
KM9_998.2
KM10_017
KM11_104
KM22_205
KM45_853
KM48_871.2
KMII_061.2
KMVI_067
KMVIII_069.2
.52
KM23_900
KM_6
KM9_998
KM21_037.2
KM47_866.2
.62
KM48_871
KMI_058
KMIII_062
KMIII_062.2
KMVIII_069
KM10_883
KM10_883.2
KM2_699
KM2_699.2
KM14_888
KM15_889
KM16_890
KM17_892
KM20_895
KM5_990
KM16_114
KM21_037
KM47_866
KMII_061
KMIV_063
1
.93
KM3_306
KM6_309
KM19_317
KM19_317.2
.72
RYA1177.2
KM12_312
RYA803.2
KM1_304.2
KM2_305
.67
KM7_310
KM7_310.2
KM14_314.2
KM15_315.2
.92
KM3_306.2
KM6_309.2
.99
BKS1012.2
KM2_305.2
KM11_311.2
KM12_312.2
KM18_316.2
BKS1012
.61
RYA1177
KM18_316
KM21_319.2
RYA803
RYA1129
.5
KM13_313.2
KM14_314
KM15_315
.72
KM11_311
KM20_318.2
.8
RYA1129.2
KM1_304
KM13_313
KM20_318
KM21_319
ENV264
VNS222
VNS222.2
1
ENV264.2
1
Virginia rail
0.0040
